# Pregnancy complications, substance abuse, and prenatal care predict birthweight in adolescent mothers

**DOI:** 10.1186/s13690-021-00642-z

**Published:** 2021-07-29

**Authors:** Miriam Hacker, Christine Firk, Kerstin Konrad, Kerstin Paschke, Joseph Neulen, Beate Herpertz-Dahlmann, Brigitte Dahmen

**Affiliations:** 1 Department of Child and Adolescent Psychiatry, Psychosomatics and Psychotherapy, University Hospital RWTH Aachen University, Neuenhofer Weg 21, Aachen, Germany; 2 Department of Anaesthesia and Critical Care , Municipal Clinic of Karlsruhe , Karlsruhe, Germany; 3 Child Neuropsychology Section, Department of Child and Adolescent Psychiatry, Psychosomatics and Psychotherapy, University Hospital RWTH Aachen University, Aachen, Germany; 4 Catholic University of Applied Sciences, Aachen, Germany; 5 JARA-Brain Institute II, Molecular Neuroscience and Neuroimaging, RWTH Aachen & Research Centre Juelich, Aachen, Germany; 6 German Center for Addiction Research in Childhood and Adolescence, University Hospital Hamburg- Eppendorf, Hamburg University, Hamburg, Germany; 7 Department of Gynecological Endocrinology and Reproductive Medicine, University Hospital RWTH Aachen University, Aachen, Germany

**Keywords:** Adolescent pregnancy, Teenage mothers, Low birthweight, Pregnancy complications, Prenatal care, Perinatal prevention

## Abstract

**Background:**

Reduced birthweight is associated with adverse physical and mental health outcomes later in life. Children of adolescent mothers are at higher risk for reduced birthweight. The current study aimed to identify the key risk factors affecting birthweight in a well-characterized sample of adolescent mothers to inform preventive public health efforts.

**Methods:**

Sixty-four adolescent mothers (≤ 21 years of age) provided detailed data on pregnancy, birth and psychosocial risk. Separate regression analyses with (1) birthweight and (2) low birthweight (LBW) as outcomes, and pregnancy complications, prenatal care, maternal age, substance abuse during pregnancy, socioeconomic risk, stressful life events and the child’s sex as independent variables were conducted. Exploratively, a receiver operating characteristic (ROC) analysis was performed to investigate the quality of the discriminatory power of the risk factors.

**Results:**

The following variables explained variance in birthweight significantly: prenatal care attendance (*p* = .006), pregnancy complications (*p* = .006), and maternal substance abuse during pregnancy (*p* = .044). Prenatal care attendance (*p* = .023) and complications during pregnancy (*p* = .027) were identified as significant contributors to LBW. Substance abuse (*p* = .013), pregnancy complications (*p* = .022), and prenatal care attendance (*p* = .044) showed reasonable accuracy in predicting low birthweight in the ROC analysis.

**Conclusions:**

Among high-risk adolescent mothers, both biological factors, such as pregnancy complications, and behavioural factors amenable to intervention, such as substance abuse and insufficient prenatal care, seem to contribute to reduced birthweight in their children, a predisposing factor for poorer health outcomes later in life. More tailored intervention programmes targeting the specific needs of this high-risk group are needed.

## Significance

*What is already known?*

Teenage motherhood entails developmental risks for mother and child. Previous research showed that teenage mothers less often attain proper education, have fewer social resources, and engage more often in risky health behaviour compared to adult mothers, and their children have a lower birthweight and are at risk for developmental adversity.

*What this study adds?*

Out of multiple potential risks, this study identified prenatal care attendance, substance abuse and pregnancy complications as significant risks for lower birthweight in a well-described sample of adolescent mothers. These findings will inform public health prevention and intervention strategies for pregnant adolescents and help to improve conditions for their children.

## Background

Previous research has emphasized the important effects of the course of pregnancy on later child mental health development (for a review, see e.g. [1]). An inadequate intrauterine environment is associated with lower birthweight in children, which is linked to adverse physical and mental health outcomes later in life [2]. Lower birthweight is associated with a significantly higher risk for cardiovascular diseases [3] and psychiatric disorders [4]. In addition, birthweight has been shown to be associated with cognitive performance: Even mild variations in birthweight between monozygotic twin pairs have been linked to differences in postnatal intelligence persisting into late adolescence [5]. One factor associated with lower birthweight of the newborn across countries is young age of the mother [6]. In welfare societies, teenage mothers are a small and heterogeneous, but often particularly burdened group of mothers [7]. To develop better prevention and intervention strategies for their children, it seems necessary to identify the risk factors that strongly affect the course of pregnancy and the child’s birthweight among teenage mothers. In the following, we will first report findings on maternal risk factors for lower birthweight for mothers of all ages. Then, we will discuss studies that directly addressed this issue in adolescent mothers, which will guide the selection of variables of interest in the present study.

A strong risk factor associated with reduced birthweight and low birth weight (LBW, < 2.500 g) is the occurrence of *medical complications during pregnancy* [8]. Concerning adolescent mothers, the findings regarding perinatal complications are ambiguous. Most studies report a higher proportion of premature births in adolescent mothers than in adult mothers [9], but also lower rates of caesarean sections, gestational diabetes, and instrumentally assisted births [10]. Azevedo et al. reported that abortion, pregnancy-induced hypertension, urinary tract infections, and premature rupture of membranes were the most frequently described complications in studies relating to pregnancy outcomes in adolescent mothers [11]. With regard to the effects on birthweight in adolescent mothers, there is a gap in the literature: studies investigating medical events during pregnancy in adolescents compared with pregnancy in adults have focused mainly on the rates of complications but lack a thorough investigation of how these events affect birth outcomes among adolescent mothers.

*Substance abuse* during pregnancy is strongly associated with reduced birthweight: Maternal nicotine abuse during pregnancy leads to lower birthweight – an average decrease of at least 200 g [12]. A significantly lower birthweight was also found to be related to the consumption of alcohol [13] and cannabinoids [14] during pregnancy. Substance abuse during pregnancy is more common in adolescent than in adult women [15]. Furthermore, substance abuse before the age of 12 is associated with a significantly higher risk for teen pregnancy [16]. Additionally, the level of substance abuse prior to conception seems to continue throughout pregnancy in adolescents [17], which is a risk factor for lower birthweight.

*Socioeconomic disadvantage* was found to be associated with lower birthweight [18]. However, many studies investigating socioeconomic factors and birthweight use neighborhood characteristics instead of the individual’s socioeconomic conditions (e.g., [19]). Although teenage mothers are a more heterogeneous group than adult mothers, adolescent motherhood is associated with lower socioeconomic resources in general [20], which might lead to adverse effects on the intrauterine environment and thus lower birthweight.

In a similar notion, current literature suggests that *stressful life events* during pregnancy such as financial problems, but also divorce or moving are associated with lower birthweight [21], which adolescent mothers might be more prone towards because of a comparatively instable living situation with less resources [22].

Not attending *prenatal care* or attending a lower number of prenatal care appointments is associated with increased risk for lower birthweight and fetal and neonatal deaths [23]. Adolescent mothers also make less use of prenatal appointments [24], which suggests that this factor could also influence birth outcome in adolescent mothers negatively.

To the best of our knowledge, there is a gap in the literature regarding key factors associated with adverse birth outcomes in adolescent mothers. Such knowledge could be informative for developing targeted public health prevention and intervention programs. Thus, the aim of our study was to assess the most important obstetrical and psychosocial factors throughout the course of pregnancy to identify the key risk factors associated with lower birthweight in a sample of well-characterized adolescent mothers.

## Methods

### Study design

In this study, a subset of the baseline data of a randomized-controlled longitudinal intervention study in adolescent mothers and their children (Registered with the German Clinical Trial Registry, Number DRKS00004409, [25]) was used. The study was approved by the local ethics committee and undertaken according to the Declaration of Helsinki and Good Clinical Practice (GCP) regulations. All participants and/or legal guardians gave written informed consent before enrolment in the study. Participants were remunerated for their participation and their travel expenses. During the first baseline visit (children’s age: 3 to 6 months), data were collected retrospectively by assessing the participant’s history and reviewing the available medical records regarding the course of the pregnancy, prenatal care attendance, perinatal outcome, socioeconomic risks, stressful life events, and substance abuse. Data collection took place before the intervention started; thus, the current data were unaffected by and unrelated to any study intervention.

### Participants

Our sample consisted of adolescent mother-child dyads (n = 64) (≤ 21 years at the time of birth). Participants were recruited through advertisements and leaflets distributed in the local youth welfare system, obstetric clinics, gynecology and midwife practices, and pediatrician practices in the locality. Mother-child dyads could be included if the children (1) were born as a singleton after the 35th gestational week, (2) did not display any signs of an inherited syndrome and (3) had no significant somatic disorder. Mothers with illicit substance abuse disorders, severe psychiatric disorders, or suicidality were excluded from participation [25].

### Measures

#### Pregnancy and birth

The adolescent mothers and/or the legal guardians were interviewed regarding their attendance of prenatal care appointments, complications during pregnancy, birthweight (in grams), umbilical cord pH, and mode of delivery (spontaneous, caesarean section, instrumental vaginal); these data were confirmed by checking the available medical records, e.g., the German “Mutterpass”, an expectant mother’s record of prenatal and natal care. The following events were considered as complications: gestational diabetes, preeclampsia, premature labor, premature bleeding, hyperemesis gravidarum, intrauterine growth restriction, cervical insufficiency, urinary stasis and urinary tract infections, placental insufficiency, gestational hypertension, and imminent abortion. If any of these events occurred, a score of “1” was given, and the scores were summed to yield a total score. Complete data on pregnancy and birth outcomes were available for n = 63 participants, data on prenatal care appointments were available for n = 59 participants, and data on the umbilical cord pH were available for *n* = 61 participants (see Table 1).

**Table 1:** Descriptive data on pregnancy and birth characteristics

	Adolescent group *n* = 63	Adult group *n* = 35	t/X²	*p*-value	Larger cohort studies	
**Characteristics**						
Maternal age at birth (years)	18.6 ± 2.0	29.8 ± 2.7	-21.2	*< 0.001*		
Gender of child			2.904	.*088*		
female	49 %	31 %			*48.72 %*^*5*^	
male	51 %	67 %			*51.27 %*^*5*^	
**Pregnancy and birth**						
Number of prenatal care appointments	12 ± 3.61	12 ± 1.94	.530	.*597*	*16.5 ± 4.7*^*1*^*11.34*^*5*^	
Detection of pregnancy (gestational week)	8.73 ± 5.06	6.31 ± 2.11	3.251	.*002*		
Number of medical complications during pregnancy	0.56 ± 0.74	0.20 ± 0.41	3.083	.*003*		
Gestational diabetes	3.2 %	2.9 %			*2.0 %*^*2*^, *5.90 %*^*5*^	
Pre-eclampsia	4.8 %	0 %			*3.8 %*^*4*,^, *3.0 %*^*1*^	
Premature labour	7.9 %	0 %			*1.87 %*^*5*^	
Antepartum bleeding	4.8 %	5.7 %			*5,3 %*^*6*^, *1.21 %*^*5*^	
Cervical insufficiency	12.7 %	2.9 %			*1.32 %*^*5*^	
Urinary stasis and urinary tract infections	7.9 %	0 %			*0.1 %*^*1*^, *0.34 %*^*5*^	
Placental insufficiency	6.3 %	0 %			*0.78 %*^*5*^	
Hyperemesis gravidarum	3.2 %	5.7 %			*0.29 %*^*5*^, *0.8 %*^*7*^	
Gestational hypertension	1.6 %	0 %			*1.33 %*^*5*^	
Intrauterine growth restriction	3.2 %	0 %				
Imminent abortion	0 %	2.9 %				
Birthweight (g)	3202.14 ± 544.50	3517.57 ± 485.33	-2.85	.*005*	*3,457 (± 497)*^*6*^	
Low birthweight (LBW) (≤ 2,500 g)	11.1 %	0 %	4.19	.*041*	*4.3 %*^*3*^, *7.03 %*^*5*^	
Umbilical cord pH	7.27 ± 0.08	7.27 ± 0.06	− 0.18	*0.859*		
Mode of delivery			1.34	*0.512*		
Spontaneous	71.4 %	62.9 %			68.02 %^5^	
Cesarean section	23.8 %	34.3 %			24.70 %^5^	
Instrumental vaginal	4.8 %	2.9 %			7.21 %^5^	

^1^Leppalahti et al., 2013: Based on 51,142 Finish women aged 25–29 years[7] ^2^Huy et al., 2012: Based on 186,818 German women aged 25–29 years [26] ^3^Rebhan et al., 2009: Based on 3,092 German women (> 18years) [27] ^4^ Abalos et al., 2013: Based on 1,093,782 European women [28] ^5^IQTIG Institute for Quality Assurance and Transparency in Health Care, federal evaluation for the year of registration 2017 - obstetrics, based on 761,481 German mothers [29] ^6^McCormack, R., et al., 2008, based on 21,685 women aged 18–35 years[30] ^7^Vikanes et al., 2008, based on 23,640 mothers from Western Europe [31] ^8^Diemert et al., 2016, based on 200 German women > 18 years (Exclusion obstetrical risk factors) [32] - In most cases, the larger cohort studies also included prematurely born infants or infants with congenital anomalies.

#### Psychosocial risk

The adolescent mothers and/or legal guardians were debriefed regarding demographic data. Educational problems, financial problems and living as a single-parent family were assessed using dichotomous coding; thus, a cumulative risk score between 0 (no risk) and 3 (high risk) was calculated to assess individual socioeconomic risk (see also [33]). Furthermore, whether the pregnancy had been planned was also assessed. Substance abuse during pregnancy (alcohol, cannabinoids, nicotine, and/or other drugs) was assessed retrospectively in personal interviews. Alcohol was quantified in glasses per week, cannabinoids were quantified in joints per month, nicotine was quantified in cigarettes per day, and other drugs were quantified in portions per month. To account for the abuse of multiple substances, a sum score was calculated using the following classification: nicotine: 0 cigarettes/d = 0, 1–4 cigarettes/d = 1, 5–9 cigarettes/day = 2, 10–14 cigarettes/d = 3, ≥ 15 cigarettes/d = 4; alcohol: 0 glasses/wk = 0, 1–3 glasses/wk = 1, 4–6 glasses/wk = 2, ≥ 7 glasses/wk = 3; and cannabinoids: 0 joints/month = 0, 1–4 joints/month = 1, 5–8 joints/month = 2, ≥ 9 joints/month = 3. The scores across substances were added to yield the substance sum score (following the procedure by [12]). Furthermore, data on stressful life events within the last year during the time of pregnancy were collected, including the following: death of a family member/loved one, own severe disease, serious disease of a family member, divorce/separation from the partner, loss of job/apprenticeship, severe change in the social environment and moving/relocation. If any of these events occurred, a score of “1” was given, and these scores were summed to obtain a total score. Complete data on psychosocial risk and stressful life events were available for *n* = 63 mother-child dyads (see Table 2).

**Table 2:** Descriptive data on psychosocial risks and substance abuse

	Adolescent group *n* = 63	Adult group *n *= 35	t/X²	*p*-value	Larger cohort studies
**Psychosocial risk**					
Socioeconomic risk score	1.58 ± 0.89	0.17 ± 0.51	9.6	*< 0.001*	
Stressful life events (any)	90.5 %	60 %	12.9	.*001*	
Number of stressful life events	2.9 ± 1.57	1.43 ± 1.27	4.8	*< 0.001*	
Death of family member or loved one	20.6 %	14.3 %			
Severe own illness	11.1 %	2.9 %			
Severe illness family member	12.7 %	14.3 %			
Breakup of relationship	31.7 %	5.7 %			
Loss of job/apprenticeship	15.9 %	2.9 %			*3.45–3.72 %*^*3*^
Severe changes in social environment	39.7 %	8.6 %			*3.45–3.72 %*^*3*^
Relocation	81.0 %	34.3 %			
Planned pregnancy	17.5 %	82.9 %	39.8	*< 0.001*	
Substance abuse during pregnancy (any)	46 %	5.7 %	16.9	*< 0.001*	
Substance dose score	1.08 ± 1.32	0.11 ± 0.47	5.2	*< 0.001*	
Alcohol	3.1 %	0 %			15.3–19.4 %^1^ 0.2 %^2^
*glasses/week*					
* 0*	*96.8 %*	*100 %*			
* 1–3*	*1.6 %*	*-*			
* 4–6*	*1.6 %*	*-*			
* ≥ 7*	*-*	*-*			
Cannabis	1.6 %	0 %			3.85 %^4^
*joints/month*					
* 0*	*98.4 %*	*100 %*			
* 1–4*	*-*	*-*			
* 5–8*	*1.6 %*	*-*			
* ≥ 9*	*-*	*-*			
Nicotine	45.3 %	5.7 %			6.5–9.2 %^1^, 7.0 %^2^
*cigarettes/day*					
* 0*	*54.0 %*	*94.3 %*			
* 1–4*	*9.5 %*	*-*			
* 5–9*	*23.8 %*	*5.7 %*			
* 10–14*	*7.9 %*	*-*			
* ≥ 15*	*4.8 %*	*-*			
Other drugs	0 %	0 %			

^1^Rebhan et al., 2009: Based on 3,069 German mothers (> 18years) [27] ^2^Leppalahti et al., 2013: Based on 51,142 Finish mothers aged 25–29 years[7] ^3^IQTIG Institute for Quality Assurance and Transparency in Health Care, federal evaluation for the year of registration 2017 - obstetrics, based on 761,481 German mothers [29] ^4^Saurel-Cubizolles et al., 2014: based on 8,620 French mothers aged 25–34 years [34]

### Statistical analysis

All statistical analyses were performed with SPSS v25 (IBM Corp., Armonk, New York, USA). For the purpose of assessing the risks of the adolescent mothers, the descriptive data were exploratively compared with data of a group of adult mothers (*n* = 35; 25–35 years at the time of birth, same inclusion/exclusion criteria). Chi-squared tests, Mann-Whitney U-tests and independent samples t-tests were performed. Additionally, the results of previous larger cohort studies regarding similar risk factors were identified and are provided for comparison (see Table 1). To identify the variables with the strongest impact on birth outcome, a linear regression using likelihood backward stepwise selection was run with birthweight as the dependent variable and the socioeconomic risk score, the child’s sex, maternal age, medical complications during pregnancy, substance abuse, prenatal care and stressful life events during pregnancy as independent variables. As most studies investigating birth outcomes in adolescent mothers used LBW as an outcome variable, we conducted an additional logistic regression using LBW as a binary outcome to provide better comparability. Likelihood backward selection and the same dependent variables were used in this analysis. Exploratively, a receiver operating characteristic (ROC) analysis was performed to identify cutoff values as well as the specificity and sensitivity of the risk factors for LBW in adolescent mothers. LBW functioned as a state variable, while maternal substance abuse, pregnancy complications, prenatal care attendance (coded inversely, as an increase should function as a protective factor against LBW), and psychosocial stress were added as test variables. One infant had a birthweight of exactly 2500 g. All calculations regarding LBW were performed with both the inclusion and exclusion of this infant in the LBW group. Because the results remained the same, this infant was put into the LBW group for power purposes.

## Results

### Descriptives

The data are presented in Tables 1 and 2. Adolescent mothers differed significantly from adult mothers regarding pregnancy complications, and birthweight. Attendance and number of prenatal care appointments as well as umbilical cord pH or mode of delivery did not differ significantly between the groups (see Table 1). Socioeconomic risk differed significantly between the groups (see Table 2). Adolescent mothers also reported a higher number and a higher incidence of stressful life events than adult mothers. The pregnancy was significantly more often planned in the adult group. The adolescent mothers reported significantly higher substance abuse during pregnancy than adult mothers.

### Factors associated with variance of birthweight in adolescent mothers

The results of the backward selection method of linear regression revealed that, the following predictors remained in the model and explained a significant proportion of the variance ( F(3,55) = 7.26, *R*²=0.28, *p = .001*): occurrence of complications during pregnancy (b=-0.33, T(3,55)=-2.86, *p = .006*), prenatal care attendance (b = 0.33, T(3,55) = 2.84, *p = .006*), and maternal substance abuse during pregnancy (b=-0.24, T(3,55) = -2.06, *p = .044*).

### Factors associated with LBW in adolescent mothers

In the binary logistic regression model, prenatal care (*p = .023*) and complications during pregnancy (*p = .027*) explained 69.9 % of the variance (Nagelkerke’s R²=0.699) in LBW; this finding was statistically significant (*p* < .001). Maternal substance abuse and psychosocial stress remained marginally significant in the model (see Table 3). With each additional pregnancy complication, the odds of LBW increased by a factor of 24.95 (96.1 %), CI [1.45; 428.95], although this finding must be interpreted with caution because of the large CI. With each additional attended prenatal care appointment, the odds of LBW decreased by a factor of 0.48 (32.4 %), CI [0.26; 0.90].

**Table 3:** Binary logistic regression results predicting LBW

	Regression coefficient B	Standard Error	exp (B)	Wald	df	*p*
**Dependent variable LBW**						
** Maternal substance abuse**	0.862	0.470	2.368	3.360	1	.*067*
** Complications during pregnancy**	3.217	1.415	24.949	4.913	1	.*027*
** Prenatal care attendance**	- 0.731	0.321	0.481	5.180	1	.*023*
** Psychosocial stress**	-1.184	0.672	0.306	3.104	1	.*078*

### Receiver Operating Characteristics Analysis

**Fig. 1:**
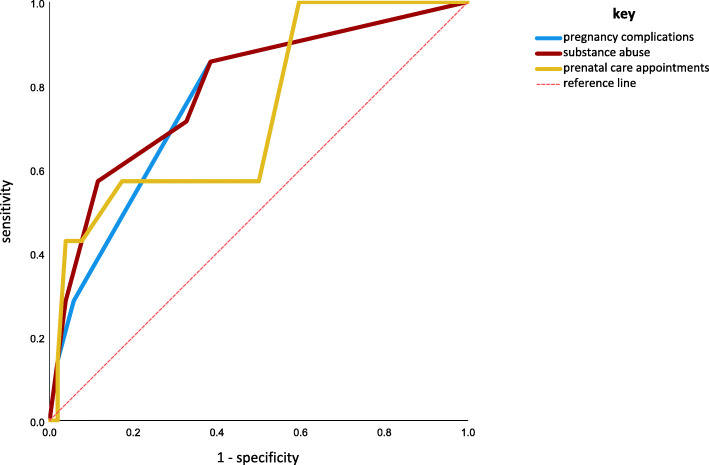
Receiver Operating Characteristic Curves for pregnancy complications, substance abuse, and prenatal care for the detection of LBW

Substance abuse (area under the receiver operating characteristic curve (AUC): 0.79, *p* = .013), pregnancy complications (AUC: 0.77, *p* = .022), and prenatal care attendance (AUC: 0.74, *p* = .044) all showed reasonable accuracy in predicting LBW in the group of adolescent mothers. Regarding cut-off values, even a low level of substance abuse (< 5 cigarettes per day) or one pregnancy complication showed a high sensitivity (86 %) and sufficient specificity (62 %) for predicting LBW in adolescent mothers. The optimal cut-off for the number of prenatal care appointments was more than 13 with a rather low sensitivity of 57 %, but a high specificity (83 %) for predicting LBW in adolescent mothers (see Fig. 1).

## Discussion

To our knowledge, this is the first study to investigate comprehensively the effects of biological, behavioral and social risk factors during pregnancy on birthweight in a well-characterized sample of adolescent mothers. We found that pregnancy complications, prenatal care attendance, and substance abuse during pregnancy all contributed independently and significantly to the lower birthweight of term-born healthy newborns of adolescent mothers. In addition, we identified the frequency of prenatal care and pregnancy complications as significant determinants of LBW in singletons of adolescent mothers. As birthweight is an important determinant of physical [3] and mental [2] health later in life, the factors leading to reduced birthweight in otherwise healthy children of adolescent mothers are highly relevant.

Regarding substance abuse, our finding of nearly half of the adolescents reporting non-illicit substance abuse during pregnancy is in accordance with the literature [17, 20]. The finding of a significant impact of substance abuse such as nicotine, cannabis and alcohol use [12–14] on birthweight is consistent with current findings in adult mothers. Substance abuse increases the risk of adverse events during pregnancy, and this risk could be a stronger target for preventive efforts. A recent study demonstrated that smoking in adolescence in general mediates the association between low family socioeconomic status (SES) and low offspring birthweight with teen smoking being linked to prenatal smoking [18]. This emphasizes the importance of public health efforts for smoking prevention in teens especially from a low family SES background. Intensive counselling and support regarding cessation programmes for adolescent mothers should be provided at the time of pregnancy detection to mitigate the higher risk for this detrimental behaviour among pregnant adolescents. Secondary prevention in the context of substance abuse in pregnancy might occur through a stronger interplay between social workers, midwives and nurses, with gynaecologists, child and adult psychiatrists, and paediatric and maternity wards “bridging” across different health domains. A promising example of this multidisciplinary prevention service, the so-called “Familieambulatoriet” (FA), has been implemented and evaluated in Norway [24]. It has a focus on mothers with substance abuse and mental health disorders, and participation in this service reduced small-for-gestational-age rates in premature newborns [24].

The mean number of prenatal care appointments utilized by the adolescent mothers did not differ from the adult mothers or the average number in Germany [29]. Recent literature reports a lower rate of prenatal care attendance among adolescent mothers than among older mothers [35] or more often insufficient prenatal care (less than half of the recommended number of appointments [7]). Designated prenatal care might also differ across countries due to the different health care systems or the percentage of the insured, which affects the comparability of studies, as suggested by Wong and colleagues [15]. The current study was performed in Germany, where a general health care system covers prenatal care irrespective of the number of appointments. Therefore, none of the mothers had to reimburse additional prenatal care appointments or were restricted from scheduling appointments because of financial resources. However, prenatal care appointments of the adolescent mothers ranged from only a few appointments (lowest count of 3) up to a maximum of 23 appointments. In our study, a higher number of prenatal care appointments was as a protective factor against low birthweight in adolescent mothers. It can be hypothesized that the adolescent mothers who took part in more frequent prenatal visits also had a lower number of additional risk factors; however, there was no association between the number of appointments and other risk factors (data not shown). Similar to substance abuse, prenatal care presents a factor that can be targeted for prevention and intervention. A recent Cochrane review concluded that receiving incentives for attending prenatal care resulted in more frequent attendance among pregnant women [36]. Attending a dedicated prenatal clinic service seems to be associated with a higher birthweight and a higher rate of spontaneous vaginal deliveries in pregnant adolescents [37]. However, further research on effective interventions to engage adolescent mothers in sufficient prenatal care is needed.

In our study, adolescents had more medical complications during pregnancy than the adult group, primarily cervical insufficiency and urinary tract complications were more frequent. This finding is partially in line with a recent review summarizing complications in adolescent pregnancy [11]. Identifying pregnancy complications as explaining variance in birthweight and LBW in term-born, otherwise healthy children emphasizes the impact of these factors among adolescent mothers. Additionally, the ROC analysis suggested that any one complication during prenatal care visits should raise concerns to increase surveillance for other risks in adolescent mothers. To our knowledge, this is the first study to identify pregnancy complications as a main factor in birthweight among adolescent mothers in a welfare society. Nevertheless, the literature about pregnancy complications in adolescent mothers is still scarce, and preventive aspects other than medical interventions alone might have a stronger impact on improving birthweight.

The group of adolescent mothers had a high socioeconomic risk (see Table 2) with 58.7 % scoring on at least 2 of the 3 risk factors. The elevated socioeconomic risk in our group of adolescent mothers is in line with previous studies [20]. We calculated an individual socioeconomic risk score comprising different aspects (education, finance and living as a single parent) for each of our participants. This is a strength of our study, considering that other studies often used neighbourhood characteristics as a representation of SES (e.g., [19]). However, socioeconomic risk did not predict the child´s birthweight in our study. As shown by Kane and colleagues [18], the effect of SES on birthweight might be mediated by other social risk factors such as prenatal smoking.

The occurrence of stressful life events during pregnancy did not explain the variance in birthweight, also pregnancy stress has been associated with birthweight in adult mothers in previous studies [21]. This difference may be explained by two reasons. First, the stressful life events were evaluated for the last year, including the first postpartum months. Second, the life events were not “pregnancy-specific”, and some studies have suggested that pregnancy-specific life events have a stronger impact on birthweight than stress in general [22].

Our study has some limitations. First, the sample size was small, which suggests that our findings should be interpreted with caution: The small sample size also impeded a more thorough investigation of each risk factor, e.g., the separate effects of the different pregnancy complications, due to power constraints. Nonetheless, our findings show that our sample – despite its small size – can be assumed to be representative of adolescent mothers in industrialized nations, as well-known risk factors were also present in our participants. Future studies should be conducted in larger samples. Second, maternal psychosocial stress and substance abuse were only retrospectively assessed and based on the mother’s self-report, which might have been amenable to reporting bias. Third, adolescent mothers with children born prematurely or with congenital disorders were excluded from our study. Prematurity is often associated with lower birthweight in adolescent mothers [11]. On the other hand, we think that this limitation might also present a strength of our study in that, even in healthy term-born newborns, the identified risk factors explained a significant proportion of the variance in birthweight, which provides even more reason to increase prevention efforts.

## Conclusions

Altogether, the presented findings make the following contributions to previous work in the field. We determined that the number of pregnancy complications, the number of prenatal care appointments and the level of substance abuse are key factors that have an impact on birthweight in term-born newborns of adolescent mothers. Birthweight is a predictor of physical and mental health over the lifespan and is a good indication of the need for early support, particularly for burdened groups in society. Our findings call for increased awareness among gynaecologists, paediatricians, midwives, child psychiatrists, and the youth welfare system regarding the risks and challenges of adolescent pregnancy. Interdisciplinary prevention and intervention efforts combining different health domains with a focus on the aforementioned risks are recommended for adolescent mothers in welfare societies.

## Data Availability

The datasets analysed during the current study are not publicly available due to the following reasons which hamper uncontrolled data sharing: Much of the data are biometric data of connected mother-infant-dyads, the sample size is small, and the participants were recruited locally over a defined period of time. The fully anonymized data are, of course, available from the corresponding author on reasonable request.

## Abbreviations

LBW: Low birth weight
ROC: Receiver operating characteristic
AUC: Area under the curve
GCP: Good clinical practice
CI: Confidence interval
